# Improving accountability through alignment: the role of academic health science centres and networks in England

**DOI:** 10.1186/1472-6963-14-24

**Published:** 2014-01-20

**Authors:** Pavel V Ovseiko, Axel Heitmueller, Pauline Allen, Stephen M Davies, Glenn Wells, Gary A Ford, Ara Darzi, Alastair M Buchan

**Affiliations:** 1 Medical Sciences Division, University of Oxford, John Radcliffe Hospital, Oxford OX3 9DU, UK; 2 Imperial College Health Partners, London, UK; 3 London School of Hygiene and Tropical Medicine, London, UK; 4 Addenbrooke’s Charitable Trust, Cambridge, UK; 5 Oxford University Hospitals NHS Trust, Oxford, UK; 6 Oxford Academic Health Science Network, Oxford, UK; 7 University of Oxford, Oxford, UK; 8 Imperial College London, London, UK; 9 Imperial College Healthcare NHS Trust, London, UK

**Keywords:** Accountability, Alignment, Collaboration, Partnership, University medical school, Teaching hospital, Academic-clinical relationships, Tripartite mission, Academic Health Science Centre (AHSC), Academic Health Science Network (AHSN)

## Abstract

**Background:**

As in many countries around the world, there are high expectations on academic health science centres and networks in England to provide high-quality care, innovative research, and world-class education, while also supporting wealth creation and economic growth. Meeting these expectations increasingly depends on partnership working between university medical schools and teaching hospitals, as well as other healthcare providers. However, academic-clinical relationships in England are still characterised by the “unlinked partners” model, whereby universities and their partner teaching hospitals are neither fiscally nor structurally linked, creating bifurcating accountabilities to various government and public agencies.

**Discussion:**

This article focuses on accountability relationships in universities and teaching hospitals, as well as other healthcare providers that form core constituent parts of academic health science centres and networks. The authors analyse accountability for the tripartite mission of patient care, research, and education, using a four-fold typology of accountability relationships, which distinguishes between hierarchical (bureaucratic) accountability, legal accountability, professional accountability, and political accountability. Examples from North West London suggest that a number of mechanisms can be used to improve accountability for the tripartite mission through alignment, but that the simple creation of academic health science centres and networks is probably not sufficient.

**Summary:**

At the heart of the challenge for academic health science centres and networks is the separation of accountabilities for patient care, research, and education in different government departments. Given that a fundamental top-down system redesign is now extremely unlikely, local academic and clinical leaders face the challenge of aligning their institutions as a matter of priority in order to improve accountability for the tripartite mission from the bottom up. It remains to be seen which alignment mechanisms are most effective, and whether they are strong enough to counter the separation of accountabilities for the tripartite mission at the national level, the on-going structural fragmentation of the health system in England, and the unprecedented financial challenges that it faces. Future research should focus on determining the comparative effectiveness of different alignment mechanisms, developing standardised metrics and key performance indicators, evaluating and assessing academic health science centres and networks, and empirically addressing leadership issues.

## Background

As in many countries around the world, there are high expectations on academic health science centres and networks in England to provide high-quality care, innovative research, and world-class education, while also supporting wealth creation and economic growth [1-16]. An academic health science centre (AHSC), also known in different countries as an academic health centre (AHC), academic medical centre (AMC), or university medical centre (UMC) is not a single institution, but “a constellation of functions and organizations committed to improving the health of patients and populations through the integration of their roles in research, education, and patient care” [17]. An AHSC is comprised of a “medical school, one or more other health profession schools or programs (such as allied health, dentistry, graduate studies, nursing, pharmacy, public health, veterinary medicine), and one or more owned or affiliated teaching hospitals or health systems” [18]. An AHSC is usually nested within an academic health science network (AHSN), which shares the AHSC’s commitment to improving the health of patients and populations through research, education, and patient care, but co-ordinates an even greater number of functions and organisations to ensure the speedy adoption and diffusion of innovation across a large number of organisations [9-12]. Thus, meeting the expectations for AHSCs or AHSNs increasingly depends on partnership working between university medical schools, teaching hospitals, and other healthcare providers in integrating their roles in research, education, and patient care.

### The “unlinked partners” model of academic-clinical relationships

For its many strengths and outstanding features – such as universal access to healthcare free at the point of delivery, publicly-funded research and education, national workforce planning, and subsidised tuition fees for medical students – the National Health Service (NHS) in England is not without weaknesses. Namely, it is not well linked to various components of academic medicine.

Teaching and research intensive hospitals (hereafter teaching hospitals) and medical schools are run by separate organisations, and those teaching hospitals that belong to different NHS trusts are separate from each other. NHS trusts are statutory public bodies that operate hospitals and specialised health centres; they are answerable to the UK government’s Department of Health through the NHS hierarchy and are funded almost entirely from general taxation.

Teaching hospitals are neither structurally integrated with primary care, nor with social care. While secondary, tertiary, and mental health care is provided by NHS trusts, primary care is provided by general practitioners (GPs), who operate as independent contractors for the NHS and are not part of its organisational structure. Likewise, social services are provided by independent local authorities funded partly by central government grants and partly by local taxes and revenues.

**Figure 1:**
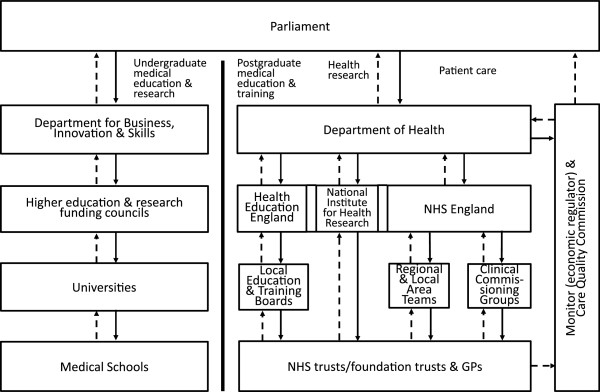
**The “unlinked partners” model of academic-clinical relationships in England, 2014.** The left and right panels represent funding and accountability relationships in the academic and clinical enterprises of AHSCs and AHSNs, respectively. Solid arrowed lines () indicate major funding flows; arrowed dash lines () indicate accountability relationships. Adapted from: Ovseiko et al. [7] and Department of Health: Equity and Excellence: Liberating the NHS. London: Department of Health; 2010.

Academic-clinical relationships in England are characterised by the “unlinked partners” model [19] (Figure 1), whereby medical schools and their partner teaching hospitals are not structurally integrated and operate under separate governance arrangements [7,19]. All medical schools are now part of universities – independent self-governing institutions, which are funded approximately equally by public and private sources [20]. Unlike many AHSCs in North America, medical schools in England do not own or govern faculty practice plans, which allow medically-qualified faculty to undertake clinical practice in owned or affiliated teaching hospitals and use the arising patient care revenue to cross-subsidise research and education in medical schools. Moreover, neither medical school deans, nor teaching hospital chief executive officers (CEOs), report to each other.

As a result of the “unlinked partners” model, university medical schools and their partner healthcare providers employ disparate finance and performance reporting metrics and indicators, no joint executive authority exists over the academic and clinical missions spanning the totality of patient care, research, and education, there is no joint staff appraisal and performance review process, and organisational cultures across the academic and clinical enterprises differ significantly [21].

At the root of these arrangements is funding from and accountability to separate government departments for higher education and health. The British government operates on the principle of “ministerial responsibility”, under which each minister is accountable for the actions of his or her own department. This system creates strong internal accountability, but also creates barriers to cross-departmental working [22].

Recent years have seen innovations in governance intended to overcome the disadvantages to academic medicine caused by this bifurcating accountability. The most common model has been the intermediate organisation, straddling the boundary between universities and NHS institutions. There has been, as yet, no systematic evaluation of the effect of these innovations, but there is growing evidence based on expert opinion and case studies of new and fruitful dynamics in some cases [4,7,8,13,15,23].

### Current policy context

Two aspects of the current policy context in England are likely to further militate against an environment that is supportive of academic medicine. One is the unprecedented financial challenges for the NHS in the broader context of growing demand and sustained constraint in public expenditure. Another is the recent re-organisation of the NHS arising from the Health and Social Care Act 2012.

#### Unprecedented financial challenges

It should be borne in mind that the English NHS is almost entirely tax-funded. Resources are allocated to providers of care through a quasi-market mechanism, whereby public monies are allocated to state-owned or authorised commissioning organisations, which contract with providing organisations to deliver care to NHS patients. According to those now leading the NHS in England, there will be a £60 billion per annum gap between funding and requirements by 2025 [24]. More independent analysis also predicts funding shortfalls on an unprecedented scale [25].

In this situation, given the doctrine of ministerial responsibility, it can be predicted that the main consideration when deciding upon funding cuts will be to minimise impact on the primary missions of departments, i.e. health in the case of the Department of Health and teaching and research in the case of the Department for Business, Innovation and Skills [26]. The extent to which each sector supports the other in the co-production of the tripartite mission of patient care, research, and education may prove to be a secondary consideration.

#### Health and Social Care Act 2012

Although the regional perspective has been historically important for the development of academic medicine in England [27], the recent re-organisation of the NHS arising from the Health and Social Care Act 2012 has led to a system in which no organisation has a clear mandate for strategic planning at the regional level. The relevant aspects of the re-organisation are the following:

First, responsibility for commissioning health care has been transferred to two types of organisation. At the local level, these are newly-formed clinical commissioning groups (CCGs) led by GPs, which replace managerially-led primary care trusts. At the national level, NHS England (formerly called the NHS Commissioning Board) is responsible both for authorisation and oversight of CCGs, and also for commissioning, through its regional and local area teams, of a wide range of services, including many specialist services provided in teaching hospitals.

Second, all NHS providers, including teaching hospitals, will have to become “NHS foundation trusts” (FTs), or join other FTs through mergers or takeovers. FTs are more autonomous NHS organisations, over which the Department of Health has no direct control. They allow better accountability to local communities through the new layer of governance that exists in all FTs – the council of governors representing local community interests. The accelerated conversion of teaching hospitals into FTs increases competition in the NHS quasi-market, but also makes collaboration more difficult because “their [FTs’] regulatory regime requires them to place organisational survival before collaboration” [28].

Third, entry into the NHS quasi-market of independent (non-NHS owned) providers of care to NHS patients is being further encouraged, so that there may be a wider variety of types of organisations providing care, although it is very unlikely that there will be any non-NHS owned teaching hospitals in England.

Finally, economic regulation of the NHS quasi-market will now be undertaken by the arm’s-length body, Monitor, in conjunction with the Office of Fair Trading. The Health and Social Care Act 2012 imposes an obligation on the Secretary of State (i.e. the government minister), NHS England, Monitor, and CCGs to promote research, but there is no obligation on providers to carry out research.

Despite the potential negative consequences of the reorganisation of the NHS, the increased autonomy of providers could be beneficial to research. Whilst the obligations on the NHS to promote research and use evidence arising from research are universal, the capacity to undertake research varies across England. The Health and Social Care Act 2012 empowers local NHS organisations to both promote and, importantly, financially support research. With this local knowledge and focus, existing partnerships can bring about a closer alignment, and where they do not currently exist, resources can be made available to support their creation. Moreover, the Health and Social Care Act 2012 raises the profile of research and its importance to the NHS. Research is now mentioned at the beginning of the Act, obligations regarding research are placed on Monitor and, as mentioned above, local clinical commissioning groups not only have duties placed on them, but explicit permission to use resources at their disposal to support research. Legislation concerning research in general, therefore, has created a permissive environment that provides institutions with a higher degree of freedom to create structures and relationships to deliver world-class research.

#### Designation of AHSCs and AHSNs

The government, as well as local health economies, have long recognised that the right balance between autonomy and partnership has to be found to make the most of the NHS quasi-market. In several policy initiatives over the past years, the government challenged local academic and clinical leaders to increase academic-clinical collaboration through alignment within the constraints of the existing system of accountability relationships.

In 2009, the government officially designated five academic-clinical partnerships as England’s first AHSCs to foster medical innovation and high-quality care through closer partnership between universities and teaching hospitals [7]. A number of local health economies in London subsequently set up wider partnerships to diffuse innovations and improve care across many local NHS trusts, as well as primary care and public health providers, with AHSCs nested within these partnerships [8,11,29].

In 2011, the government deemed it desirable to have such wider partnerships established everywhere in England and subsequently invited interested parties to apply for designation as AHSNs. These were envisaged as “a unique opportunity to align education, clinical research, informatics, innovation, training & education and healthcare delivery,” with the goal being “to improve patient and population health outcomes by translating research into practice and developing and implementing integrated health care services” [10].

In 2013, the government designated new AHSNs with the role of “increas[ing] strategic alignment of NHS providers and their university partners, specifically in world-class research, health education and patient care [in order to] improve health and healthcare delivery including through increased translation of discoveries from basic science into benefits for patients” [30]. The government designation was meant to enhance the prestige of the designated organisations and to provide AHSNs with start-up grants, but the government deliberately did not set out new structural and governance arrangements for AHSCs and AHSNs, recognising that the complexity of the challenges to be addressed by them, in particular AHSNs, required a new level of freedom to innovate.

Structures created to accommodate changes in the NHS to align patient care with research and education are likely to be strongest at the local level. As discussed, there are many factors at the national level, from legislation to the policies derived from it, that create innate challenges for NHS/university partnerships. However, at local and now potentially regional levels, relationships and partnerships can be created that focus on local issues and develop working practices that are independent of national regimes. This is particularly true where legal strategic partnerships are created, as the nature of these arrangements is likely to survive further changes to the healthcare sector by future governments.

### A framework for the analysis of accountability relationships

The “unlinked partners” model of academic-clinical relationships and the ongoing structural fragmentation of the NHS result in a complex system of accountability relationships in the universities and healthcare providers that form the core constituent parts of AHSCs and AHSNs, which ultimately influences the capacity of academic and clinical leaders to make strategic choices conducive to the advancement of both their organisations and the public good.

In conceptual terms, accountability is “a strategy for managing expectations” [31]. The need for such a strategy arises because the public has the right to know what universities and teaching hospitals are doing in order to meet public expectations for innovative health technology, high-quality care, and wealth generation, while also responding to economic demands for efficiency savings and funding cuts during economic downturns.

In operational terms, “accountability involves relationships in which an individual or agency is held to answer for performance and involves some delegation of authority to act” [32]. Through a system of accountability relationships involving multiple government and public agencies, the public delegates authority to act to medical school deans and teaching hospital CEOs, and then holds them answerable for their performance. In turn, the deans and CEOs face multiple and potentially conflicting expectations and demands from staff members responsible for different aspects of patient care, research, and education that may not fully align with each other, as well as from healthcare commissioners and higher education and research funders who employ disparate performance metrics and indicators.

The complex system of accountability relationships in AHSCs and AHSNs becomes clearer if we employ a framework of accountability relationships, which was originally developed by Romzek and Dubnick for the United States’ National Aeronautics and Space Administration (NASA) [31] and then adapted for the analysis of medical reform [32]. It distinguishes between four types of accountability [31,32]:

● *hierarchical (bureaucratic) accountability* concerns supervisory control by higher authorities;

● *legal accountability* concerns compliance with laws and contractual obligations;

● *professional accountability* concerns compliance with of the accepted norms and practices in one’s profession or peer group;

● *political accountability* concerns responsiveness to key stakeholders and constituents.

In what follows, we apply Romzek and Dubnick’s framework to analyse accountability for the tripartite mission of patient care, research, and education in universities and teaching hospitals that form core constituent parts of AHSCs and AHSNs. Then, using examples from the AHSC and the AHSN in North West London, we outline a number of mechanisms that academic and clinical leaders can employ locally to improve accountability for the tripartite mission through alignment. Finally, we argue that to avoid AHSCs’ and AHSNs’ crumbling under the weight of potentially conflicting expectations and demands, the current accountability systems need to be aligned as a matter of priority.

## Discussion

### Accountability for patient care

Under the current model of academic-clinical relationships, all hierarchical, legal, and political accountability for patient care is concentrated in the NHS, and universities are free from any formal accountability for the provision of patient care. Inadvertently, this limits the ability of universities to increase the involvement of medical school faculty in the provision of high-quality patient care, dissemination of the latest technology, and translational research with immediate patient benefits.

On the one hand, universities’ funding through the Department for Business, Innovation and Skills’ higher education and research funding councils incentivises them to employ basic scientists, who are less costly to employ and may be perceived to be more productive than physician-scientists (called “clinical academics” in England) because basic scientists do not need to take time off from their scientific work to provide patient care, and they tend to publish in more prestigious scientific journals [33].

On the other hand, under the adverse conditions of the economic downturn, NHS hospitals are dis-incentivised from employing physician-scientists because they take time off from their clinical work to do academic work, and the NHS inadequately reimburses hospitals for innovation and high-end specialist services that are usually provided by physician-scientists. The current Payment by Results tariff system for reimbursing NHS trusts is based on an average price across England and thus can tip teaching hospitals that provide complex high-quality services into deficit, and can damage their ability to work with industry on the development of new treatments [34]. Moreover, the recent proposals of the Department of Health to abolish Clinical Excellence Awards – which compensate outstanding physician-scientists, who devote substantial time to research and teaching, with longer hours and a lesser income than full-time physicians – risks undermining the global competitiveness of English AHSCs and AHSNs by making physician-scientist careers in England unattractive [35].

Yet, funding from the National Institute of Health Research (NIHR) offers a potential mechanism and incentives for universities to increase the involvement of medical school faculty in the provision of high-quality patient care, dissemination of the latest technology, and translational research with immediate patient benefits. Much of NIHR funding is either provided to universities, or to NHS/university partnerships. The NIHR holds both universities and NHS organisations accountable for this funding through contractual terms. Therefore, this begins to align accountabilities for research and patient care, at least from a potential impact and improvement perspective.

***Hierarchical and legal accountabilities*** of university staff with clinical responsibilities are managed through the NHS. In order to undertake clinical practice, research, and teaching, university-employed academic physician-scientists must secure honorary contracts with teaching hospitals. Honorary contracts deal with matters of indemnity and accountability. They allow university faculty to undertake clinical practice in the NHS setting, usually on an unpaid basis. Universities and teaching hospitals are not fiscally linked because they receive funding from and are answerable to different government departments – the Department for Business, Innovation and Skills and the Department of Health, respectively. Instead, they operate the knock-for-knock arrangements, whereby university-employed academic physicians provide patient care to NHS patients without charging the NHS, and in return NHS staff provide teaching to medical students without charging universities [7].

Neither universities nor teaching hospitals measure the actual quantities of patient care and teaching provided as part of these arrangements. This is, however, beginning to change. It is now not unusual for individuals within a NHS trust to have specific portions of their overall salary directly linked to job plans identifying specific clinical, or research time. Moreover, the NHS and university partners will each contribute to the salary, therefore formally sharing the investment in research. Despite this change, there remains a separation of hierarchical and legal accountability between the NHS and universities, which makes it challenging for some academic and clinical partners to agree and conduct joint annual appraisal and performance review processes for staff working across organisational boundaries [36].

***Professional accountability*** rests with professional regulatory bodies – the General Medical Council (GMC) and the relevant specialty medical royal colleges and faculties. They are responsible for setting and monitoring standards of medical education and training, registering qualified doctors, revalidation, enforcing medical codes of conduct, upholding professional values, issuing evidence-based clinical guidelines, and participating in the appointments committees of NHS organisations. For quality and safety of care, physicians are also accountable to each other through clinical governance and risk management groups in their organisations [37].

***Political accountability*** is mediated by the democratic election process, through which elected politicians hold top civil servants in the Department of Health to account according to the mandate and expectations of the public. In practice, however, it is hard to distinguish between hierarchical and political accountability because of the hierarchical nature of the NHS in England [38]. Despite government’s numerous reforms, which attempted to decrease ministers’ micromanagement of the NHS and involve patients and the public in decisions about local health services, accountability to patients and local communities is still limited and exists in parallel with centralised political accountability [39]. For example, although many hospitals have now converted their status to more autonomous NHS foundation trusts (FTs) evidence to date indicates that nationally-set objectives and targets still play a large role in FTs’ decision-making [40]. Moreover, local clinical commissioning groups (CCGs) are subject to a large degree of national oversight by NHS England [41,42].

### Accountability for research

In recent years, great advances have been made in enabling research across universities and the NHS through the implementation of the recommendations of Sir David Cooksey’s review of UK research funding and the subsequent establishment of the NIHR in England [43]. Yet, the English system for the funding of research remains notably pluralistic, and both universities and the NHS maintain separate systems of research governance, creating a challenge for integration at the institutional level of AHSCs and AHSNs.

***Hierarchical accountability*** for research involves parallel structures for universities and NHS bodies, which overlap and sometimes combine in the field of clinical research. Each sector has its own structures for research governance, and these sit within a wider spectrum of research regulation and governance. Directives from the European Union, most notably the Clinical Trials Directive (2001/20/EC), add a further level to the structures for hierarchical accountability. Concern about the negative effect of medical research regulation on the national economy has led to the establishment in 2011 of a Health Research Authority, with a mission to streamline the regulatory process and expedite research.

A further characteristic of the environment for medical research in England is the rich mix of public and private actors on both the payer and the provider side [44]. For individual NHS/university partnerships, this complex regulatory and funding environment has the potential to create costly and duplicative research management and governance structures. To try and mitigate this, universities and teaching hospitals are attempting to create joint structures that seek to better align management though integration of working practices and co-location of staff.

Establishing joint research offices is becoming more common in England. However, this approach requires significant investment, both financially and politically, to overcome the inherent difficulties of working across organisations. The desire to achieve economies of scale via the combination of these structures is countered by separate accountabilities and risk aversion, but there is some room for pragmatic adaptation. For example, the NHS is able to act as “research sponsor” for clinical studies led by university staff as it is more able to manage the risk of clinical negligence liabilities. Although a joint research office can be successful at dealing with governance and financial issues, it cannot in itself develop a research strategy. It remains a problem that many NHS trusts do not have a clear research strategy and often have no clear framework for innovation undertaken outside a research setting.

***Legal accountability*** is overly complex and split disproportionally between universities and teaching hospitals. While the majority of clinical research is designed, carried out, and reported by university-employed academic physician-scientists in collaboration with NHS-employed physicians and nurses, hospital managers are legally responsible for the approval of clinical research. Historically, because research metrics have not been included in the annual appraisal and performance review of hospital managers, they tend to overemphasise patient safety risks and underemphasise patient benefits from research. However, the government has identified the need to see a significant improvement in establishing clinical trials in the NHS and, therefore, many NHS organisations are now including research metrics in appraisal at divisional management level, and several AHSNs have made this a priority area. A better understanding and acceptance of risk would benefit research in general and promote patient access to innovations.

For government research funding, universities are accountable to the Department for Business, Innovation and Skills and its arm’s-length research funding councils, and NHS trusts are accountable to the Department of Health along with its arm’s length bodies, particularly those involved in regulation. For public and private research funding, universities and NHS trusts are accountable to medical research charities, industry, and other funders on the basis of contractual obligations.

***Professional accountability*** mainly concerns research integrity and is devolved to individual universities and voluntary professional associations. Academic scientists are accountable to their peers via university research integrity and ethics committees, internal and external peer review, and national and international professional associations in any given research area. Academic physician-scientists are also accountable to professional regulatory bodies, but universities and professional associations do not always have coherent research integrity policies. A recent parliamentary inquiry found “the general oversight of research integrity in the UK to be unsatisfactory” [45] and reiterated an earlier recommendation to establish a national oversight body for research integrity [46]. Most NHS trusts that have research integrity policies in relation to patient care accept accountability only to those professional associations that provide a licence to practise and do not accept accountability to voluntary professional associations that do not provide such a licence.

***Political accountability*** reflects the fragmentation of hierarchical accountabilities between universities and the NHS. Because at the national level political accountability for the NHS and universities is located in different government departments, there is no unified stewardship, oversight and scrutiny of all stages of health research by elected officials. At the local level, many universities and teaching hospitals establish strategic alliances with medical research charities, partnerships with industry, and patient and public involvement forums, through which they can be directly accountable to research users and participants, but this form of accountability is still in its infancy.

One particular mechanism that is most successful in improving accountability for research to the community is research funding provided by the NIHR and the Department of Health. These two funders have established processes and commit significant resources to promote the involvement of patients and the public in research [47]. This funding provides academic and clinical partners with incentives to involve patients, the public, and carers in setting research priorities and, where appropriate, helping direct research activity. It is also essential that clinicians and researchers are involved in any priority-setting exercise to establish feasibility of the research and also to recognise the importance to their patients of the topic of that research.

### Accountability for education and training

A recent parliamentary inquiry concluded that the current education and training system is “too complex… and that accountability is poor” [48]. The fragmentation and separation of accountability for undergraduate medical education, junior doctor training, specialty training, and academic training between university and NHS partners results in a broken continuum of knowledge and experience for tomorrow’s doctors. Contrary to the calls of academic and clinical leaders to allow universities to assume responsibility for postgraduate education and training [49], the latest government reforms have missed an opportunity to reduce the separation of undergraduate and postgraduate education and training in universities and the NHS respectively, and have in fact increased it.

***Hierarchical accountability*** for undergraduate education and training lies with universities. The Higher Education Funding Council for England – which is an arm’s-length funding council of the Department for Business, Innovation and Skills – plans and funds university places for medical undergraduates in accordance with the workforce requirements of the NHS. Universities run medical schools, provide non-clinical teaching, and award degrees.

Hierarchical accountability for postgraduate education and training lies with postgraduate deaneries that, under the latest government reforms, have been incorporated into Local Education and Training Boards (LETBs). The latter are statutory committees of a new NHS statutory health authority – Health Education England – that plans and funds clinical training posts. As part of LETBs, postgraduate deaneries commission and quality-manage specialty training provided by teaching hospitals and general practitioners, and academic physician-scientist training provided by universities [50].

***Legal accountability*** for education and training is divided between the NHS, universities, and professional regulatory bodies. An important addition is that postgraduate deaneries have to comply with the UK Employment Agencies Act because formally they are not part of the higher education sector and are instead treated by law as employment agencies. Moreover, postgraduate deaneries and teaching hospitals have to comply with European Union regulations, which often have a major impact on postgraduate education and training. For example, according to the Working Time Directive, junior doctors can work a maximum of 48 hours per week (including inactive on-call time).

***Professional accountability*** fully rests with professional regulatory bodies. Following the merger of the Postgraduate Medical Education Training Board, a government body that previously regulated postgraduate education and training, with the General Medical Council (GMC) in 2010, the medical profession became responsible for regulating all stages of medical education and training. While the GMC quality-assures education and training programmes at universities and postgraduate deaneries, medical royal colleges are responsible for setting and overseeing the specialty curricula within these programmes and for awarding qualifications and memberships.

***Political accountability*** at the national level is split between the two government departments, and thus there is no aligned stewardship, oversight and scrutiny of the entire continuum of knowledge and experience across undergraduate and postgraduate education and training. At the local level, many university medical schools and teaching hospitals assume accountability for the education and training of health professionals to the local community, region, and nation they serve [51], but it is yet to be formally established.

### How can AHSCs and AHSNs address deficits in accountability through alignment?

Although government policies to promote AHSCs and AHSNs provide academic and clinical partners with symbolic and monetary incentives to improve collaboration, the government has stopped short of reforming the existing accountability relationships to allow closer academic-clinical integration. Accountability relationships in the universities and healthcare providers that form core constituent parts of AHSCs and AHSNs remain complex and fragmented, as summarised in Table 1. Therefore, local academic and clinical leaders face the challenge of improving accountability for the tripartite mission without the prospect of a fundamental system redesign that would support fully integrated governance. There is a growing consensus on both sides of the Atlantic that, when fundamental reform is not politically feasible, the joint working of the component parts of academic medicine can be strengthened, and thus accountability for its tripartite mission improved, only through improved alignment [7,23,52,53].

**Table 1:** Accountability relationships in universities and healthcare providers that form core constituent parts of AHSCs and AHSNs

	**Universities: Medical schools**	**Healthcare providers: Hospitals and GPs**
**Hierarchical**	Department for Business, Innovation and Skills (BIS); university.	Department of Health (DH); NHS trusts/FTs; GP practices/CCGs.
**Legal**	Health research legislation and regulatory bodies [35]; higher education legislation; Quality Assurance Agency for Higher Education; undergraduate medical education and research funding grants including the DH’s National Institute for Health Research (NIHR) and contracts with BIS’s higher education and research funding councils, medical research charities, industry partners, and students.	Healthcare legislation and regulatory bodies [54]; National Audit Office; Care Quality Commission; Monitor (economic regulator); Office of Fair Trading; health research legislation and regulatory bodies [35]; postgraduate medical education and training contracts with Health Education England and Local Education and Training Boards; health service contracts between commissioners (CCGs and NHS England) and NHS trusts/FTs; national contract between GPs and NHS England; research grants and contracts with NIHR, medical research charities, and industry partners.
**Professional**	General Medical Council, medical royal colleges and faculties; Academy of Medical Sciences; national and international professional associations; research community peers; university research ethics committees.	General Medical Council; medical royal colleges and faculties; NHS medical director; NHS trust/FT medical director; NHS trust/FT clinical governance and risk management peer groups.
**Political**	Parliament; BIS; NHS and industry partners; public involved in research; local and global patient communities.	Parliament, NHS England (and through its mandate, DH); local authorities’ overview and scrutiny committees; patient and public representatives’ Healthwatch; NHS trust/FT non-executive directors; NHS FT governors and members.

Alignment of institutional accountability, goals and activities within NHS/university partnerships creates a foundation on which to build relationships with other stakeholders in the health and social care sector. While local partnerships centred around NHS trusts can most obviously influence acute care provision, they can also (if carefully constructed) bring the same academic rigour to the entire patient pathway from the promotion of health through to the management of chronic illness in the community. At its core, this model is dependent on partnerships, and the creation of AHSCs and AHSNs has the potential to greatly facilitate this model of working, promoting interaction between stakeholders from academia, the NHS, public health, local government, and industry. Importantly, clinicians and scientists can begin to work with parts of the health care sector to develop and evaluate interventions that can promote good health and prevent disease, thereby delivering patient benefit at an earlier stage, where it can have more impact.

North West London provides interesting examples to demonstrate various alignment mechanisms that academic and clinical leaders can use to improve accountability locally. It also exemplifies the importance of local initiative and leadership in pursuing local health improvements, and illustrates the contemporaneous development of a bottom-up partnership and the national AHSN scheme.

In 2009, academic and clinical partners at Imperial College London and the Imperial College Healthcare NHS Trust established the first AHSC in England to foster academic-clinical collaboration, focusing mainly on bridging the translational gap between discoveries and innovations in the lab and new treatments and ways of caring for patients in the clinic [4,7]. In 2011, a review of the AHSC conducted by one of the authors (AD) concluded that North West London would benefit from a wider partnership to drive innovation and implementation at a larger scale and a faster pace, i.e. speeding up the adoption of innovation and closing the gap between what is known to be best practice and what is actually delivered by many NHS trusts [55].

The evidence for this review was collected for administrative and policy purposes. It was based on expert advice from the review steering board, engagement with staff at the Imperial College AHSC, and discussions with wider NHS and academic stakeholders in North West London. Given that there has been no systematic evaluation of AHSCs in England, this review provided unique insights into various alignment mechanisms that can be used at Imperial College and, possibly, other AHSCs.

### Imperial College AHSC

The AHSC at Imperial College London and Imperial College Healthcare NHS Trust was not only the first AHSC in England, but it was also characterised by a unique organisational model. Whereas all other English AHSCs implemented the “joint partnership board” model, the Imperial College AHSC pursued the “joint leadership and management” model [7]. This latter model can be explained using the following three dimensions of medical school-clinical relationships derived from the US literature [56]:

● *Clinical enterprise organisation.* Unlike the clinical enterprises of the other English AHSCs, which were comprised of several independent NHS trusts, the clinical enterprise of the Imperial College AHSC was comprised of a single NHS trust. It was created as part of the AHSC project in 2007 through the merger of two acute hospital trusts, and at that time it became the largest NHS trust in England.

● *Academic-clinical enterprise integration.* Whereas the other English AHSCs created joint partnership boards for leaders from separate organisations to co-ordinate the delivery of the tripartite mission across the organisational divide, the Imperial College AHSC pursued the strategy of academic-clinical enterprise integration based on joint leadership and management appointments, such as the dean of the medical school/CEO of the clinical enterprise, and chairs of academic departments/chiefs of clinical services.

● *Authority position of the chief academic officer over the clinical enterprise.* In contrast with the other English AHSCs, where the chief academic officer (i.e., the highest-ranking official responsible for the academic mission) did not have any formal executive authority over the clinical enterprise, the joint appointment of the dean/CEO at the Imperial College AHSC provided the chief academic officer with executive authority over the clinical enterprise.

The “joint leadership and management” model proved to be highly successful in achieving a transformational shift in academic-clinical collaboration and creating the first AHSC in England in 2007 [4]. However, it did not prove to be viable in the long run due to significant financial challenges for both the academic and clinical enterprises during the economic downturn. While the academic enterprise faced the challenge of addressing changes to higher education funding streams, the clinical enterprise faced the challenge of rebalancing its finances in the face of no real terms growth of revenues from clinical services. Given that under the current “unlinked partners” model of academic-clinical relationships in England the cross-subsidisation of academic and clinical missions is impossible, the academic and clinical enterprises at the Imperial College AHSC had to face their financial challenges separately.

Following the departure of the founding dean/CEO to take up a new international role, the decision was taken to separate this appointment and, subsequently, the Imperial College AHSC moved towards the “joint partnership board” organisational model based on a formal joint working agreement and a strategic partnership board. Moreover, similar to the other English AHSCs, the Imperial College AHSC entered into an extended partnership with other NHS organisations in its catchment area, i.e. North West London. Overall, we can observe convergence in organisational models between AHSCs in England.

The evolution of the organisational model of the Imperial College AHSC has important implications for our understanding of the role of leadership in AHSCs. It is clear that in the case of the Imperial College AHSC different organisational challenges required different leadership models.

During the formation of the AHSC, the “joint leadership and management” model was required in order to bring academic and clinical partners together as an AHSC for the first time, and to signal their commitment to the principles of the AHSC. We can hypothesise that this set of challenges is better addressed by “transformational leadership” based on principles-driven work [57]. Moreover, it is plausible to assume that this set of challenges was more effectively addressed by leaders with a considerable amount of institutional knowledge and well-developed relationships with colleagues in both the academic and clinical enterprises.

During the economic downturn, the “joint partnership board” model was required in order to clearly demarcate financial challenges and to separate accountabilities for financial performance. We can hypothesise that this set of challenges is better addressed by “transactional leadership” characterised by strict performance and outcome criteria [57]. We can also assume that this set of challenges is more effectively addressed by leaders with a proven track record of successfully addressing similar challenges in different institutions. More research is needed in order to analyse these leadership issues empirically.

### Imperial College Health Partners

In parallel with changes at the Imperial College AHSC and following the recommendations of the sector review by AD, academic and clinical partners in North West London set out to create a wider partnership for the distinct purpose of the speedy adoption and diffusion of innovation. Consequently, and under the continued leadership of AD as Chair, “Imperial College Health Partners” (ICHP) was launched in June 2012 as a company limited by guarantee [29] prior to the AHSN process. It is relevant to note that personal relationships and leadership were as important in this context as the factual business case for joining the partnership, given the voluntary nature of ICHP.

ICHP comprises Imperial College and the NHS organisations of North West London, including acute hospital care, mental health, community health, and primary care (through clinical commissioning groups) [58]. Considerable effort went into engaging primary care colleagues, both as commissioners and providers, making the case for a partnership bridging the provider-commissioner split that so often tends to dominate day-to-day relationships. Members pay an annual membership fee that funds a small central executive team. The fee is the same for all members, who each have one vote on the Board, reflecting the values of the partnership of inclusiveness and equality.

The objectives of the partnership are three-fold: enabling the discovery of best practice and speedy adoption; consistent implementation of good practice; and working with industry and international partners to create wealth for the NHS. ICHP is very much a bottom-up initiative, informed, funded and led locally and voluntary and, therefore, unique in its nature and composition compared to almost all other relationships and organisations in the local health economy. At the same time as the partnership established itself, NHS England announced the establishment of AHSNs across England. Given the almost identical objectives of AHSNs and of ICHP, the partnership applied for an AHSN licence. Consequently, ICHP was designated as an AHSN in September 2013 and is still expanding its membership to ensure public health and industry are well-represented, though this has proven difficult.

In addition to the AHSC, there are other partnerships nested within ICHP that help align accountabilities for academic and clinical missions in research and patient care. The NIHR Imperial Biomedical Research Centre (BRC) [59] and two NIHR Biomedical Research Units (BRUs) [60] are bilateral NHS/university partnerships between Imperial College London and its partner NHS trusts that focus on translational biomedical research. The NIHR London (North West) Comprehensive Local Research Network (CLRN) is a multilateral partnership between a large number of NHS organisations that aims to increase participation in clinical research in all areas of disease and healthcare [61]. The NIHR Collaboration for Leadership in Applied Health Research and Care (CLAHRC) for North West London is a multilateral partnership between a large number of NHS organisations, universities, charities, and pharmaceutical companies that works to accelerate the translation of research evidence into practice across primary and secondary interfaces of care [62]. Importantly, all research and implementation projects supported by these NIHR partnerships have a strong patient and public involvement element. For example, all applications for CLAHRC funding must include plans to involve patients and the public in their research and implementation activities [63].

ICHP is more than a loose affiliation of partners, but less than a full merger in terms of accountabilities for individual organisations within the partnership. However, the inbuilt stability and the signal that this sends to external partners such as industry (partners have committed for multiple years independently of changes in government or AHSN funding, which was awarded for one year in the first instance), the frequency of meetings (the Board meets four times a year), and a strong sense of mutuality implied in the legal form of the partnership (CEOs are directors of the company with a range of responsibilities) will go some way to align incentives, build trust, and increase accountability.

The operating model is based on a small central team that is providing dedicated resources to pursue the objectives of the partnership. However, where ever possible staff from partner organisations are seconded into specific projects to ensure the necessary expertise and buy-in. All of these are seen as crucial structural components to achieve better outcomes across a fragmented sector. The award of some government funding through a successful AHSN application provides a further lever to align partners in the sector and increase the scope. But it also implies that conditions attached to the funding need to be carefully balanced with local priorities, and the constituent members of ICHP have expressed a strong desire to ensure the sustainability and identity of the original partnership.

### Examples of alignment mechanisms

Overall, the evidence which was gathered as part of the initial review of the AHSC at Imperial College London [55], discussions to create ICHP [29], and the experience of its implementation, suggest that stronger accountability would be best achieved through alignment in a number of ways at different levels of the partnership. In particular, this is realised:

#### Through leadership and management changes at the top of the partnership

First, a key principle of the partnership is an equally-represented leadership and management structure. If there is only limited cross-representation between academic and clinical organisations on their boards, it is important to create equally-represented leadership and management structures for joint strategic planning and decision-making, e.g. a strategic partnership board with a joint executive group and specialist committees. Second, some joint leadership and management appointments were made so that there are senior staff who have combined executive authority over the academic and clinical enterprises. Third, leaders are supported by a small and dedicated central management team, which is funded through membership fees as set out above.

#### Through changes to bring together staff at lower levels in the partnership

First, several of the partnership projects are or will be run through collaboration between member organisations supported by the central team. This way it is easier to bring academic and clinical staff together to focus on specific diseases and organ systems if academic departments were to map onto clinical services. Second, one of the medium-term aims is to move towards a single research strategy for the whole partnership with alignment of individual organisations’ research strategies. This can be achieved in a number of ways, such as by appointing a joint director of research, or by collocating partners’ research support functions in a joint research office. This is also something that industry has identified as a priority area. Third, fundraising efforts are being co-ordinated to focus on joint capital projects and to avoid competing for the same donors. However, this is not to replace existing activity, but to harness complementarities across the partnership where appropriate.

#### Through changes at all levels in the partnership

First, common measures of success are being identified both across all projects and for each individual project. Although academic and clinical partners use a variety of metrics and indicators to measure their separate performance, it is essential that they agree on a common framework that measures the strength of the tripartite mission in a balanced way. Second, a sense of unity and common purpose has been instilled by adopting a joint brand identity, running a joint website, circulating joint information material, and publishing a joint annual report with a common performance matrix. Third, where differences in organisational culture between academic and clinical partners form significant barriers to innovation and collaboration, specific development plans will be established at project level. Joint clinical, academic, and managerial appointments also help to overcome these barriers.

### A future research agenda

Each of these alignment mechanisms has the potential to counter the separation of accountability for patient care, research, and education at the national level by improving accountability for the tripartite mission from the bottom up. In the absence of strong evidence about the best mechanisms and their effectiveness in different settings, evaluation and assessment of AHSCs and AHSNs is essential in order to determine the comparative effectiveness of different alignment mechanisms. As more knowledge is generated, it should become possible to identify the mechanisms of greatest leverage for achieving enhanced accountability in different settings, and to see whether such mechanisms are strong enough to overcome the separation of accountability for the tripartite mission at the national level. Ultimately, academic and clinical leaders could use this knowledge to enhance the performance of their AHSCs and AHSNs locally, as well as to advocate particular reforms nationally.

While conducting evaluation and assessment of AHSCs and AHSNs, it is imperative to develop standardised metrics and key performance indicators in order to enable both national and international comparisons. Given that the overwhelming majority of patient care, research, and education in England is funded by the government and charities, they could use standardised metrics and indicators to hold AHSCs and AHSNs accountable for the quality of patient care, research, and education. Moreover, government agencies and charities could use standardised metrics and indicators to ensure value for money for the public investment in AHSCs and AHSNs through open competition between institutions. There is a need for standardised metrics and indicators not only in England, but also internationally [64]. This would allow academic and clinical leaders to accelerate performance improvements at their institutions by benchmarking and comparing performance across countries and by identifying areas of collaboration with their peers around the world.

Finally, future research should empirically address leadership issues in AHSCs and AHSNs. The current paper focused on structure rather than agency, i.e. on institutions rather than leaders. It showed that the current accountability relationships make the structural integration of universities and healthcare providers extremely unlikely and, thus, local academic and clinical leaders face the challenge of aligning their institutions within the framework of the existing sub-optimal accountability relationships. Moreover, leadership in academic-clinical settings is different from leadership in traditional hierarchical organisations: AHSC and AHSN leaders have the challenge of “leading among leaders” [65]. However, research on leadership in academic and clinical settings in England is underdeveloped. Currently, there is no empirical research on the leadership styles and characteristics of the leaders that are most effective in AHSCs and AHSNs. Also, it is currently unknown whether the most effective leaders come from inside or outside of the institution. Therefore, future research should empirically address leadership issues in AHSCs and AHSNs in order to help institutions select the right leaders, and help leaders-to-be understand what is required to succeed.

## Summary

At the heart of the challenge for AHSCs and AHSNs in England to provide high-quality care, innovative research, and world-class education, while also supporting wealth creation and economic growth, is the separation of accountabilities for the tripartite mission of patient care, research, and education in different government departments. It prevents universities and healthcare providers from cross-subsidising academic and clinical missions and from creating fully integrated AHSCs and AHSNs. Given that a fundamental top-down system redesign to allow the structural integration of universities and healthcare providers is extremely unlikely, local academic and clinical leaders face the challenge of aligning their institutions as a matter of priority in order to improve accountability for the tripartite mission from the bottom up.

It remains to be seen which alignment mechanisms are most effective and whether they are strong enough to counter the separation of accountabilities for the tripartite mission of academic medicine at the national level, and the unprecedented financial challenges that it faces. It also remains to be seen whether AHSNs, which are not yet partnerships in their own right, can achieve a level of durability that is required to establish them as credible organisations. Future research should focus on determining the comparative effectiveness of different alignment mechanisms, developing standardised metrics and key performance indicators, evaluating and assessing academic health science centres and networks, and empirically addressing leadership issues.

## Competing interests

AD recommended the adoption of the AHSC model in England in his role as Parliamentary Under-Secretary of State in the Department of Health, conducted the review of the Imperial College AHSC in his role as Chair of the AHSC Steering Board, and took up the role of Chair of Imperial College Health Partners until October 2013. AH led the development of Imperial College Health Partners in his role as Director of Strategy and Business Development at the Chelsea and Westminster Hospital NHS Foundation Trust, and took up the role of Director of Strategy and Commerce of Imperial College Health Partners. AMB participated in the review of the Imperial College AHSC in his role as Dean of Medicine and Head of the Medical Sciences Division of the University of Oxford. GAF is Chief Executive Officer of the Oxford Academic Health Science Network. PVO, PA, SMD, and GW declare that they have no competing interests.

## Authors’ contributions

AMB jointly conceived of the paper with PVO, participated in its design, and helped to draft the manuscript. PVO undertook the literature review and led the writing of the paper. AH, PA, SMD, GW, and GAF co-wrote parts of the paper and critically commented on drafts. AD contributed ideas arising from the review of the Imperial College AHSC, participated in the co-ordination of the paper, and critically commented on drafts. All authors read and approved the final version of the manuscript.

## Pre-publication history

The pre-publication history for this paper can be accessed here:

http://www.biomedcentral.com/1472-6963/14/24/prepub
